# 580. Lessons from a Burn Mass Casualty Incident at a Single ABA-Verified Burn Center

**DOI:** 10.1093/jbcr/irag033.343

**Published:** 2026-04-06

**Authors:** Maeva Adoumie, Brianna L Collie, Sean Carrington, Ann Higgins-Alford, Joyce I Kaufman, Shevonne S Satahoo, Louis R Pizano, Carl I Schulman

**Affiliations:** University of Miami Jackson Memorial Hospital, Miami, FL; University of Miami Jackson Memorial Hospital, Miami, FL; Jackson Memorial Hospital, Miami, FL; Jackson Memorial Hospital, Miami, FL; Miami Burn Center, Miami, FL; Miami Burn Center, Miami, FL; Miami Burn Center, Miami, FL; Miami Burn Center, Miami, FL

## Abstract

**Introduction:**

Mass casualty incidents are often the result of penetrating or blunt traumatic injuries, rather than burn injuries. Real-world experience with burn mass casualties is therefore limited. Burn injuries require distinct triage and initial management, making burn mass casualties especially unique. The objective of this study is to describe the lessons learned and the opportunities for improvement after a burn mass casualty event, with 11 patients transferred after a single event, to an ABA verified Burn center, all arriving within 1 hour of each other.

**Methods:**

A review of the initial triage and treatment of patients during the mass casualty event was performed. The treating physicians and staff involved in the burn mass casualty incident were debriefed and opportunities for improvement were discussed. The attending physician in charge made notes as to the successes and failures during the mass casualty event, with recommendations for improvement.

**Results:**

11 patients were seen and treated as part of the event. Age ranged from 5 to 41 years old. 91% (10/11) patients had burns >10% TBSA, 73% (8/11) patients had burns >20%, and 4 were intubated prior to or shortly after arrival. The event took place overnight on a hospital observed holiday. The receiving hospital activated its Trauma Local MCI Response, bringing additional staff from the floors and ICU. All in-house surgical trainees were recruited to assist. In addition to the initial trauma assessment, the burn specific issues were the need to debride and dress so many large burn wounds simultaneously, the need for good photo documentation and burn mapping to guide resuscitation, the need for continued monitoring of adequate resuscitation while waiting for floor or ICU beds, and the need for managing multiple intubations, escharotomies, and the OR simultaneously.

**Conclusions:**

No patients suffered from under or over resuscitation in the first 24 to 72 hours. Several areas were noted for improvement in subsequent events. First, a single individual at the intern level needs to be responsible for ensuring all burns are photo documented, and all burn maps are completed (the photo/map czar). There needs to be a single person who makes sure all the dressings are performed according to the plan of the attending physician (the dressing czar). There also needs to be a single person who monitors all the resuscitations, focusing on proper fluid rates titrated according to urine output (the fluids czar). The burn attending must refrain from participating in any of these tasks and must focus on triage and management decisions as the mass casualty evolves, eventually performing the more invasive surgical procedures. Overall, the early hours in burn care are essential for resuscitation and patient care, thus learning from these mass casualty events can be vital in future incidents.

**Applicability of Research to Practice:**

This situation is a possibility for any Burn Center to experience, so these lessons learned can be invaluable for future events.

**Funding for the study:**

N/A.

